# An intergenerational program based on psycho-motor activity promotes well-being and interaction between preschool children and older adults: results of a process and outcome evaluation study in Austria

**DOI:** 10.1186/s12889-019-6572-0

**Published:** 2019-03-01

**Authors:** Erika Mosor, Karin Waldherr, Ingvild Kjeken, Maisa Omara, Valentin Ritschl, Veronika Pinter-Theiss, Josef Smolen, Ursula Hübel, Tanja Stamm

**Affiliations:** 1 Section for Outcomes Research, Center for Medical Statistics, Informatics, and Intelligent Systems, Medical University of Vienna, Spitalgasse 23, 1090 Vienna, Austria; 2 FernFH Distance-Learning University of Applied Sciences, Zulingergasse 4, 2700 Wiener Neustadt, Austria; 3 National Advisory Unit on Rehabilitation in Rheumatology, Diakonhjemmet Hospital, Pb 23 Vinderen, 0319 Oslo, Norway; 4 AKMÖ - Aktionskreis Motopädagogik Österreich, Döblinger Hauptstr. 7a/2/43, 1190 Vienna, Austria; 5 Division of Rheumatology, Department of Internal Medicine III, Medical University of Vienna, Währinger Gürtel 18-20, 1090 Vienna, Austria; 6 Wiener Gesundheitsförderung – WiG, Gesunde Stadt – Gesunde Organisationen, Treustraße 35 – 43/6/1, 1200 Vienna, Austria

**Keywords:** Older adults, Health promotion, Intergenerational relations, Evaluation, Well-being, Psychosocial, Public health

## Abstract

**Background:**

Limited evidence exists for intergenerational interventions to promote health and well-being in older adults and preschool children. We therefore aimed to evaluate the implementation, feasibility and outcome of an intergenerational health promotion program based on psycho-motor activity.

**Methods:**

A multicenter mixed-methods study with preschool children and older adults as equivalent target-groups, and professionals and parents as additional informants was conducted in Austria. The study included a needs assessment, a pilot phase with a formative process evaluation and a subsequent rollout phase to evaluate the outcome and the adapted processes of the intervention program. To analyze the qualitative data, a modified form of the framework method was applied. Quantitative data were collected with a time-sampling method and were analyzed using descriptive and inferential statistical procedures.

**Results:**

One hundred ninety-six participants (93 older adults [54 to 96 years old, 83% female], 78 children [2 to 7 years old, 58% female], 13 professionals and 12 parents) from 16 institutions (eight kindergartens and eight geriatric facilities) were included in the study. The qualitative process evaluation revealed several aspects for improvement of the intervention program. Well-being as measured by observing spontaneous intergenerational contacts (*p* < 0.001) and facial expressions (effect size r = 0.34; *p* < 0.001) showed a significant increase between the rollout baseline and follow-up assessments.

**Conclusions:**

Professionals in geriatric institutions and kindergartens could facilitate interactions between members of the different generations by offering an intergenerational intervention program based on psycho-motor activities in the future.

**Electronic supplementary material:**

The online version of this article (10.1186/s12889-019-6572-0) contains supplementary material, which is available to authorized users.

## Background

Promoting health and well-being in older adults is an essential public health issue nowadays and will be even more important in the future due to the continuously increasing number of older adults [1, 2]. Physical activity, emotional and social interaction and active participation turned out to be effective in preventing falls, treating chronic diseases and disabilities, as well as enhancing well-being in older adults [3–5].

Different generations and the contact between older adults and children are valuable resources in our society. On the one hand older adults can share their knowledge, support and inspire younger generations; on the other hand children and grandchildren are essential for older generations, e.g. by offering new insights and perspectives, using new technologies which could assist older adults and representing the “young part” in the life of a human being. As family patterns and community structures have changed considerably during the last decades, older adults and children often experience reduced intergenerational contact [6–8]. Social contact and sharing a common goal has long been considered to be one of the most effective strategies for improving inter-group relations and reducing prejudices among different groups [9]. Aday and colleagues [10] identified three success factors for intergenerational contact: talking with peers, gathering concrete experience with the other generation and expanding the knowledge about the other group.

Intergenerational programs and interventions connect younger and older generations to foster positive experiences, learning, and appropriate socialization for both young and old [11, 12]. Thus, they could be important future approaches in the public health area. In a recent review about the impact of intergenerational programs on children and older adults, Gualano et al. (2018) pointed out that a wide range of shared activities like playing games, acting creatively, singing and dancing facilitated interaction and well-being in the participants [12].

Psycho-motor activities in an intergenerational context aim at supporting health and well-being of an individual by providing pleasurable social interaction and promoting physical activity. They are based on a holistic view of people, regarding each individual as a unit of physical, emotional and cognitive realities that interact with each other and the surrounding social environment [13, 14]. In order to cover a range of health issues, including physical, emotional, cognitive and social aspects in older age and young age, a psycho-motor based intervention in an intergenerational context was adopted in this study.

In recent years, a few intergenerational group programs with older adults and kindergarten children have been developed and implemented, but only few of them have been evaluated [15–17]. Evidence for the feasibility and impact of intergenerational group programs involving young children and older adults was found to be largely lacking [16, 18–20]. Furthermore, some studies focused either on the benefit for older adults only or on added values for children [19, 21–23]. To date, studies equally adressing both target groups, including different settings and large numbers of participants are rare [20, 24, 25]. Furthermore, most of the studies have not included the perspectives and perceptions of caregivers, kindergarten teachers and parents of kindergarten children, or environmental factors when conducting such an intervention [26].

In order to develop, conduct, evaluate and implement innovative programs and practices several steps are necessary. Randomized controlled trials (RCTs) are important to assess effects of interventions in medical care and in the public-health area. Prior to conducting an RCT, mixed-methods designs allow focusing on implementation and feasibility of an intervention and can be the basis for well designed RCTs [27, 28]. Furthermore, involving all relevant stakeholders throughout the duration of the study increases the acceptance and applicability of an intervention [29, 30] and makes it fit for the target groups which is a precondition for the effectiveness of an intervention.

The aim of this study was to evaluate the implementation, feasibility and outcome of an intergenerational health promotion intervention program based on psycho-motor activity in Vienna, Austria.

## Methods

### Study design

**Fig. 1:**
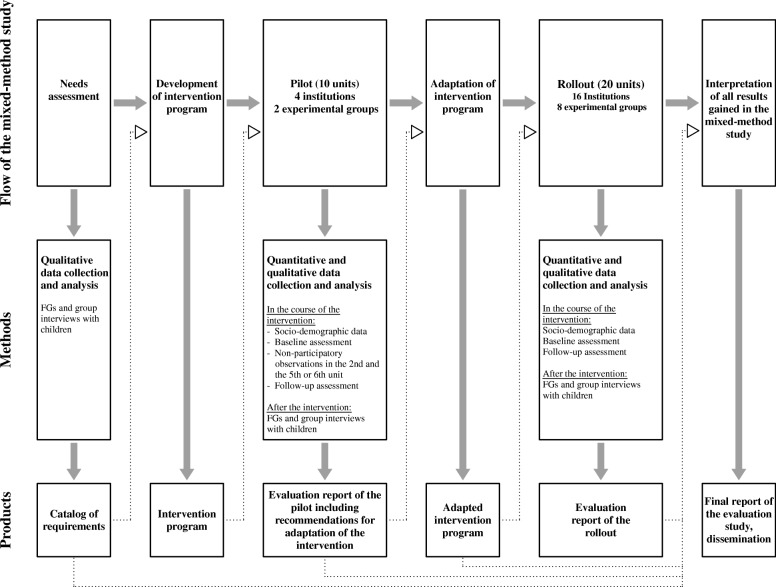
Design and flow of the study

We conducted a multicenter, mixed-methods study using an exploratory sequential design. We started with a needs assessment, targeted at all stakeholders, followed by the development of the intervention program and a pilot phase that included a qualitative process evaluation. Based on the results of the pilot phase, the intervention program was adapted and implemented in a rollout phase including an outcome evaluation as well as a final qualitative process evaluation (Fig. 1). A participatory approach was followed [31] and older adults and kindergarten children were defined as equally important target groups, participating in all steps of the evaluation study. Furthermore, the perspectives of professionals (caregivers, kindergarten teachers) and parents were explored in the course of the needs assessment and process evaluation. Moreover, professionals were actively involved in data collection and were trained to conduct the assessments for the outcome evaluation. In addition, non-participatory observations through members of the evaluation team took place during the second, as well as the fifth or sixth sessions of the pilot, to develop an understanding of the setting of interest. Qualitative and quantitative data were collected during different time-points of the study, analyzed separately and then discussed and integrated in a final report.

### Participants and institutions

A convenience sample of eight geriatric institutions and eight kindergartens in Vienna was selected to participate in this study. We considered a range of different institutions essential for ensuring rich qualitative data and an outcome evaluation that is applicable for real life settings. Subsequently, older adults and kindergarten children, as well as professionals (caregivers and kindergarten teachers) and parents were recruited by the participating institutions. Older adults aged 50+ had to be either residents in a long term care institution or visitors of a senior day-care centre. Children, between 2 and 7 years of age, were eligible to participate if they attended the kindergarten on a regular basis. Participants of both groups had to be able to join an intergenerational, psycho-motor activity group session as assessed by their caregivers. Multimorbidity, restrictions in mobility, e.g. use of a wheelchair, mild cognitive impairment and limited German language skills were no reasons for exclusion. However, older adults with severe depression, severe dementia, as well as those who were permanently in need of care were excluded from participation in the study. Participant recruitment started in October 2014 and ended in March 2016. Health professionals and kindergarten teachers were trained in recruiting eligible participants as part of a half-day training course by the evaluation team. Based on their expertise, another person was recruited as a surrogate to keep the group size constant, in case a child or older adult could not, or did not want to continue the intervention program. A balanced number of older adults and young children taking part in different psycho-motor activities was regarded as a basic requirement in the intergenerational program. Recruitment of the participants was left to the decision of the professionals working in the institutions to apply as accurately as possible the real life situation. Participants for the qualitative study were selected using a maximum variation strategy depending on the different stakeholder groups and considering the following criteria: coming from different institutions, both sexes, different functional mobility and cognitive status, different culture and ethnicity, different (former) profession and levels of training.

### Ethical consideration

The study was submitted to the responsible ethics committee and approved without further detailed review (EK 14–274-VK_NZ). Eligible participants were first asked whether they would like to participate in the study and were informed in detail about the study and the right to withdraw at any time. All participants gave oral and written informed consent. Participating children assented to the intervention program in addition to their parents’ written consent. All data were handled with strict confidentiality and were only used for research purposes.

### Intervention – Intergenerational contact through psycho-motor activity

The structured intervention program was newly developed and further adapted based on the results of the needs assessment and the pilot evaluation in the course of the study [32]. A short overview about the content of the intervention program and the activity-inducing material used is given in Additional file 1. Each session focused on intergenerational contact by means of psycho-motor activities and was facilitated by two trainers. The trainers had experience in working with children and older adults in group settings. The intervention sessions included activities that facilitated sensory, perceptive, motor and social skills by using different types of material, which should induce and be an incentive for creative psycho-motor activities, including tubes, ropes, marbles, newspaper and plastic cups (Additional file 1). The trainers suggested intergenerational activities which had to include a motor component and should facilitate the creative ideas of the participants. Besides ritualized welcome and closing procedures, participants were supported in occupying themselves with different appealing material and working together as a group. While interacting with the material, the participants were in motion and in contact with each other. The pilot phase included ten sessions which were conducted in two intergenerational groups. Each group consisted of a kindergarten (with up to seven participating children) and a senior citizen institution (with up to eight participating older adults). During the rollout phase, 20 sessions took place in eight intergenerational groups. The sessions were implemented on a weekly basis with a duration of one hour.

### Qualitative process evaluation

An extensive qualitative process evaluation took place throughout the project. The results of the needs assessment and the formative process evaluation in the pilot phase were used to develop and to refine and adapt the intervention after the pilot phase. Furthermore, the final qualitative focus group session was scheduled at the end of the rollout phase to ensure that the perspectives and needs of all stakeholders were comprehensively included until the very end of the project. Identifying points of strength and barriers concerning feasibility and sustainability of the intervention program at different time points, and carrying out appropriate adjustments are important steps for ensuring best results.

### Qualitative data collection

Qualitative methods including group interviews with children [33] and focus groups with older adults, professionals and parents were used to assess the needs for an intergenerational group activity. Furthermore, the implementation and feasibility of the intervention program were evaluated [31, 34–37]. Data collection and analyses were iterative processes and carried out in parallel [38]. During the preliminary stages of exploring the perspectives of different stakeholders on the intervention program, initial structures of interview schedules were developed. They followed a review of qualitative literature about intergenerational contact, and experiences with other intergenerational group programs. The interview questions for the needs assessment on an intergenerational group activity focused on values and preferences, as well as expectations of children and older adults, professionals and parents (Additional file 2). The interview questions for the pilot and the rollout phases focused mainly on how participants experienced the impact and sustainability of the intervention program (Additional file 3). The questions were pretested in older adults and children before conducting the focus groups and the group interviews, and were adapted according to their feedback [39].

All focus groups and group interviews were conducted in a familiar environment (either geriatric facility or kindergarten) and were chaired by trained and experienced moderators (EM, KW, TS) together with one assistant. Field notes on observations of the group interactions and the primary topics of discussion were taken during the focus groups. At the end of each focus group, a summary of what was discussed in the session was presented to the group to enable the participants to verify and amend emergent issues. All group interviews and focus groups were conducted in German, digitally recorded and transcribed verbatim. The quotations were subsequently translated by a native speaker for publication. After each data collection, a debriefing between moderator and other members of the evaluation team took place to review the course of the focus group and group interview [40].

Several strategies were used to improve and verify the trustworthiness of the qualitative data: data triangulation was achieved by interviewing different stakeholders at various sites, comparing findings to literature throughout the project duration and by including qualitative and quantitative methods of data collection [34, 41, 42]. After analyzing the qualitative data, all results were discussed with an experienced researcher (TS) who had not been involved in the analysis of the transcripts.

### Qualitative data analysis

A modified form of the framework method was applied [43]. Data analysis was facilitated by using QSR International’s NVivo 10 qualitative data analysis software [44]. Firstly, the transcripts were thoroughly read to gain an overview of the collected data. In a second step, data were divided into meaning units (defined as specific units of text, either a few words or a few sentences with a common meaning) and the concepts contained in the meaning units were identified. Concepts could refer to the main topic of a meaning unit, but a meaning unit could also contain more than one concept. A concept is a separate theoretic entity with some attributes different from other concepts. Thirdly, all concepts contained in the meaning units (lower-level concepts) were grouped under higher-level concepts and sorted into a table depicting an analytical framework. Higher-level concepts are more general and cover the attributes of the lower-level concepts. In a final step we formulated requirements (needs assessment) and feedback statements (pilot- and rollout phases) out of the higher-level concepts. An example of a meaning unit, as mentioned by a child is: *“I’m sorry that it* [the group sessions] *is over now! They* [trainers] *always brought such nice material* […]. *The hair ties were so cool. And once, Leopoldine* [older adult] *made a very cool paper airplane for me.”* Three concepts were identified within this meaning unit: emotion, interesting material, and intergenerational contact. Finally, two of them (emotion, intergenerational contact) were grouped under the higher-level concept *intergenerational contact takes place*.

### Outcome evaluation – assessments and outcomes

Basic socio-demographic information was obtained from all participants. *Well-being and active engagement* assessed through *facial expressions* was the primary outcome and *engagement/behavior*, *intergenerational interaction* and s*elf-efficacy* were the secondary outcomes. As we also included older adults with cognitive impairment, a modified form of the time sampling method [16, 45] was used to assess facial expressions and engagement/behavior. According to the standard protocols a designated amount of time, a so-called observational unit (120 s) was defined and facial expression as well as engagement/behavior were observed and recorded every ten seconds within a two minutes period of time during the most active part of the intervention. For example facial expression was assessed within 60 s according to the categories happy/smiling, neutral, lethargic or grumpy. Spontaneous intergenerational interaction (yes/no) and self-efficacy were assessed based on observation if it ever occurred at least once in a session. Self-efficacy was assessed on a 5-point grading scale ranging from 1 (very good) to 5 (not sufficient) by rating the following criteria: trying out something new, showing confidence in her/his own abilities, coping with demands and responding adequately to unexpected situations. The baseline assessment took place during the second session, which was the first unit in which children and seniors came together. The follow-up assessment took place at the final session. There were at least two assessors of the participating institutions (a health professional and a kindergarten teacher) responsible for assessing the participants. Each assessor scored only one participant’s facial expressions in a given time frame (one minute). Assessors were trained in the observation of participants and subsequent scoring during a half-day training course delivered by the evaluation team. Video sequences of similar group interventions were shown, scoring was practiced and individual feedback was given. All assessments were carried out according to the standard protocols. Furthermore, frequency of participation in the intervention program, reasons for not attending, as well as adverse events (AEs) were documented throughout the pilot and the rollout phases.

### Statistical analysis

All quantitative data were checked for accuracy and completeness and data clarification forms were sent to the study sites. Descriptive statistics were used to summarize the characteristics of the participants. Metric variables were tested for normal distribution. In case of non-normally distributed data, we depicted medians and interquartile ranges (IQR) in addition to mean values and standard deviations (SD). Nominal data were shown as absolute and relative frequencies. The outcome of the intergenerational intervention program on the older adults and the children was calculated by comparing the data at the beginning and at the end of the rollout phase and applying appropriate inferential methods for paired samples by using the “last observation carried forward” method (the missing follow-up visit value was replaced by the subject’s previously observed value at baseline). The older adults and children who were enrolled as surrogates in the rollout were assessed in the respective first session when they started to take part in the program. To determine potential significant differences for non-normally distributed variables, we used Wilcoxon Signed Rank Tests for paired data and McNemar tests for nominal data. The effect size was calculated by using the formula r = $\frac{\mathrm{Z}}{\sqrt{\mathrm{N}}}$ as suggested by Rosenthal [46], where Z is the z-value of the Wilcoxon Signed Rank Test and N is the number of observations over the two time points. As multiple tests were performed, Bonferroni correction was applied. The Bonferroni-corrected level of statistical significance was set at α = 0.001 (0.05/36 pairwise tests). The Statistical Package for Social Sciences (SPSS 24) was used for all calculations [47].

## Results

### Participant characteristics

In total, 196 participants, including 93 older adults, 78 kindergarten children, 13 professionals and 12 parents, from 16 institutions (eight kindergartens and eight geriatric facilities) took part in the present study, from November 2014 to June 2016 in Austria, Vienna. Table 1 presents the characteristics of the participants.

**Table 1:** Descriptive statistics of the study participants

Participants	Older adults	Children	Professionals	Parents
Total number of participants in the mixed-method study (*N* = 196)				
Participants *n* (%)	93 (47)	78 (40)	13 (7)	12 (6)
Gender female, *n*	77	45	13	12
Age median in years (min/max)	84 (54 to 96)	6 (2 to 7)	–	–
In the pilot (*N* = 31)				
Participants *n* (%)	15 (48)	16 (52)		
Gender female, *n*	10	10		
Age median in years (min/max)	79 (54 to 91)	6 (5 to 7)		
In the rollout (*N* = 140)				
Participants *n* (%)	78 (56)	62 (44)		
Gender female, *n*	67	35		
Age median in years (min/max)	86 (56 to 96)	5 (2 to 6)		
In the qualitative process evaluation (*N* = 73)				
Participants *n* (%)	27 (37)	21 (29)	13 (18)	12 (16)
Gender female, *n*	21	14	13	12
Age median in years (min/max)	82 (54 to 95)	6 (4 to 7)	–	–

Note: Some older adults and children participated in the intervention program AND the process evaluation. Therefore, the total number of participants in the study is less than the sum of all participants in the intervention program and process evaluation taken together. The age of the professionals and parents was not assessed

### Results of the process evaluation regarding implementation and feasibility

Eleven focus groups with older adults, professionals and parents and eight group interviews with children were conducted over the whole study period (total number of participants involved *n* = 73, Table 1). The results of the qualitative process evaluation (summarized in Table 2) revealed nine requirements for the development and implementation of an intergenerational intervention program for older adults and young children. Six main themes emerged from the data of the formative process evaluation of the pilot. They helped to adapt the program for the rollout according to the feedback of participants, professionals and parents. Finally, the results of the process evaluation at the end of the rollout phase were organized in five overarching themes supporting the results of the outcomes evaluation and pointing out the value of the developed intervention program from the perspectives of all stakeholders.

**Table 2:** Summary of the results of the qualitative process evaluation

A. Requirements (results of the needs assessment)	
Requirement	Description
1	Information about the program should be given to all stakeholders in an appropriate manner, including oral information, written material, practical examples and the possibility to ask questions before the participants decide, if they want to participate.
2	Consider the principle of voluntariness for all participants in the program.
3	Kindergarten teachers and caregivers should be present during the sessions as important reference persons for the participants (children and older adults).
4	The content of each session of the group program/intervention should be carefully planned and relate to the session(s) already completed; each session should contain rituals.
5	The trainers responsible for the group sessions should have basic information on the motor and cognitive abilities of each participants.
6	The first session of the group program should be held separately with older adults or children only to give time for introduction of the program/intervention.
7	Mobility aids would not prevent people from participating in the program; they must be stored away safely during the session or appropriately used, e.g. a wheelchair can be used by a group participant if needed; children, however are not permitted to play with mobility aids of older adults.
8	An appropriately-sized room allowing each participant enough space for the planned movement (min. space per participant was estimated with 4 m^2^), a place for changing the clothes, and a nearby toilet should be available.
9	Water should be offered, either during the session or afterwards.
B. Feedback from the pilot	
Theme	Description
Acceptance	The group program/intervention was in general well received by the participants.
Need for collaboration	Additional time should be set aside for communication between the external trainers and the involved staff of the institutions to facilitate the collaboration.
Balanced offers for old and young	The needs of the participants, both older adults and children, should be taken into account in each session. Information about the course of the session should be given in an understandable way before the start of the session.
Avoidance of waiting times	Waiting times/inactive periods of time before and during the sessions should be reduced to a minimum and the timeframe should be kept; hyperactivity resulting of boredom in some children irritated some older adults.
Availability of enough space	A room with an appropriate size is an absolute must – in very small rooms the sessions cannot be performed as planned.
Extended use of material	The used material could remain at the institutions until the next session to be used by other children and older adults who are not part of the group program/intervention.
C. Feedback from the rollout	
Theme	Description
Being different from other offers	The program was experienced as “something special and different” from usual exercise classes or other intergenerational activities.
Contact between very old and very young	Intergenerational contact takes place: the program led to a self-perceived increased intergenerational contact between very young and very old persons, in terms of quantity (more contacts, more time spent in intergenerational activities), as well as in terms of quality (understanding the needs of the respective others creates more options to achieve aims in tasks together).
Changed attitude	The program changed the attitudes towards the respective other group.
Need for supportive environment	The implementation of an intergenerational program needed a supportive environment in management and administration.
Continuing contact	Direct intergenerational contact between older adults and children has continued after the end of a session (in between two sessions) and after the end of the program.

Note: The first part (A) of the table includes the results of the first qualitative part of the study, the needs assessment; the second part (B) of the table shows results collected subsequent to the pilot phase; and part C presents findings gained after the rollout phase

#### Results of the needs assessment

At the beginning of the study, three focus groups and two group interviews with children were conducted. The needs assessment revealed nine requirements (Table 2, part A). Based on these requirements the program was developed by the trainers, the setting for conducting intergenerational group activities was defined and materials organized. One theme which came up in all groups was the principle of voluntariness (requirement 2). One older adult pointed out:

*“If it makes me angry, I will stop doing it!”*

When being asked for the basic conditions for taking part in an intergenerational group program, most participants pointed out that kindergarten teachers and caregivers should be present during the sessions as important reference persons for children and older adults, to ensure that the participants feel at ease and confident when joining the intergenerational program (requirement 3). One caregiver said:

*“We should be part of it. Older adults might get any health problems* [ … ]*, and the emotional support* [by the kindergarten teacher or caregiver] *is also important. It is us whom they know best. And we know when it gets too much for them.”*

#### Results of the pilot process evaluation

The qualitative evaluation of the pilot phase revealed six main themes, considerable and partly unexpected suggestions for improvement of the intervention program (Table 2, part B).

##### Need for collaboration

Regardless of various environmental, and personal factors, additional time should be set aside for communication between the external trainers and the involved staff of the institutions to facilitate the collaboration. One kindergarten teacher described this in the following way:

*“The communication between each other* [between kindergarten teacher and trainers] *was not always easy. It needs a lot of time to organize everything. We have to talk to the children, we have to talk to the caregivers and older adults and we have to talk to the trainers.”*

##### Avoidance of waiting times

Some seniors and caregivers were not satisfied with the local situation and/or organizational conditions. Waiting times/inactive periods of time before and during the sessions were challenging for some older adults as well as for children and therefore should be reduced to a minimum. Hyperactivity resulting of boredom in some children irritated some older adults. Two older adults referred to this with the following statements:

*“During waiting times, when children had nothing to do, some of them were getting restless and fooled around. That’s why the presence of the kindergarten teachers was important.”*

*“I don’t like to wait in the beginning* [of a group session]*. There was such an unrest and the children learned nothing from it!”*

Based on the suggestions for improvement the content of the group sessions and the organisation procedures were adapted. For the rollout the participants were informed in even more detail about the aims, procedures, other participants (either older adults or children) and the content of the intervention program. Furthermore, organisational procedures were changed in some places: one centre decided to move the place where the group sessions were held from the daycare centre for older adults to the kindergarten due to limited available space. More time was given for communication between professionals of institutions and trainers.

#### Results of the final process evaluation after the rollout

The last part of the qualitative analysis at the end of the rollout phase showed five themes with solely positive experiences of the participants and their caregivers (Table 2, part C).

##### Being different from other offers

Unexpectedly, the group sessions were experienced as “something very special and different” from usual exercise classes or other intergenerational activities.

*“I was interested in everything! There were things I’ve never seen before, I’ve never done before. It was absolutely new territory for me!”* (older adult).

*“Today, we visited the older adults again. It was very nice. We have played and danced”* (child).

A woman referred to this uniqueness of the intervention program with the following statement:

*“It was different from doing gymnastics, where the children start doing exercises and running around, and we were mostly watching them.* [ … ] *Here* [in the group sessions]*, the children worked together with us, with plastic threads, pearls and balloons”* (older adult).

##### Changed attitude

Older adults who had looked critically at limited German language skills in some children before the group sessions had started, recognized that it was no problem to communicate with these children at all. One woman expressed it this way:

*“One could also communicate in non-verbal ways with the children. You didn’t need any language when moving around and building towers. Just try it out! Nothing dangerous has happened”* (older adult).

### Quantitative assessment of the outcome

**Fig. 2:**
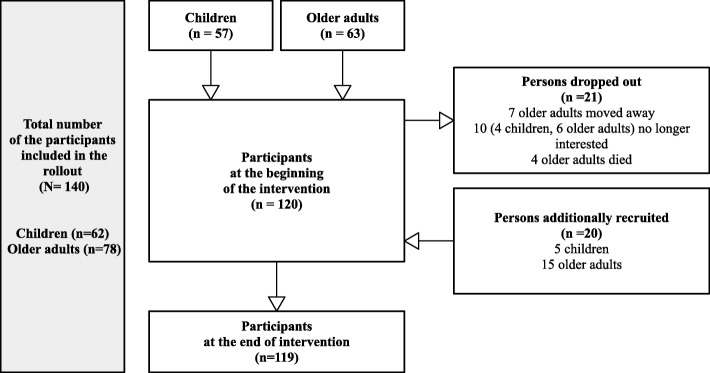
Flow of the participants in the rollout

The rollout phase started in spring 2016 with 120 participants (Fig. 2). In total, 21 persons had to stop their participation in the group sessions. To keep the group sizes constant, 20 new persons were recruited as surrogates during the period of the rollout and assessed according to the protocol. There were no significant differences at baseline in participants who took part throughout the rollout, compared to the surrogate participants (Additional file 4). Children and older adults who took part from the beginning of the rollout until the end participated on average in 16.4 sessions (SD ± 2.9) out of 20. Those who were included later on as surrogates participated on average in 11 sessions (SD ± 5.7; Mann-Whitney U-Test: *p* < 0.001).

All metric variables were non-normally distributed. The outcome evaluation of the rollout revealed a statistically significantly higher number of individuals being actively engaged with happy facial expressions (primary outcome) after the intervention when compared to baseline (Table 3; Wilcoxon Signed Rank Tests for paired data: *p* < 0.001, mean ± SD baseline 2.1 ± 1.8, mean ± SD follow-up 3.1 ± 1.7; effect size r = 0.34). When children and older adults were analyzed separately, the significant difference was confirmed in the children (mean ± SD baseline 1.6 ± 1.6; follow-up 3.3 ± 1.8; r = 0.47; *p* < 0.001). However, although a numerical difference was found with the same trend in older adults, no significant difference was observed (Table 3). Accordingly, a statistically significantly lower number of neutral facial expressions in all participants (total) and in the group of children was reported.

**Table 3:** Results of the rollout. Significance level was Bonferroni adjusted due to multiple testing

Outcome	Assessment	Categories/Items	Total baseline mean (SD) median (IQR)	Total follow-up mean (SD) median (IQR)	Total *p*-value	Older adults baseline mean (SD) median (IQR)	Older adults follow-up mean (SD) median (IQR)	Older adults *p*-value	Children baseline mean (SD) median (IQR)	Children follow-up mean (SD) median (IQR)	Children *p*-value
**Active engagement**	**Facial expression** (primary outcome)	Happy/smiling	2.1 (1.8) 2 (0 to 3)	3.1 (1.7) 3 (2 to 4)	0.001*	2.6 (1.8) 3 (1 to 4)	2.9 (1.7) 3 (2 to 4)	0.135	1.6 (1.6) 1 (0 to 3)	3.3 (1.8) 4 (2 to 5)	0.001*
		Neutral	3.6 (1.8) 3 (2 to 5)	2.7 (1.7) 3 (2 to 4)	0.001*	3.2 (1.8) 3 (2 to 5)	3.0 (1.7) 3 (2 to 4)	0.244	4.0 (1.8) 4 (3 to 6)	2.4 (1.7) 2 (1 to 4)	0.001*
		Lethargic	0.2 (0.9) 0 (0 to 0)	0.1 (0.5) 0 (0 to 0)	0.121	0.2 (0.8) 0 (0 to 0)	0.1 (0.5) 0 (0 to 0)	0.180	0.3 (1.0) 0 (0 to 0)	0.2 (0.5) 0 (0 to 0)	0.399
		Grumpy	0.1 (0.3) 0 (0 to 0)	0.1 (0.3) 0 (0 to 0)	0.931	0.0 (0.2) 0 (0 to 0)	0.0 (0.0) 0 (0 to 0)	0.317	0.1 (0.4) 0 (0 to 0)	0.1 (0.4) 0 (0 to 0)	0.589
	**Engagement/ behavior**	Participating actively	3.9 (1.7) 4 (3 to 5)	4.4 (1.6) 5 (4 to 6)	0.006	3.7 (1.8) 4 (3 to 5)	3.9 (1.6) 4 (3 to 5)	0.546	4.1 (1.7) 4 (3 to 6)	5.0 (1.5) 6 (4 to 6)	0.003
		Paying attention/listening	1.9 (1.6) 2 (0 to 3)	1.5 (1.5) 1 (0 to 2)	0.011	2.1 (1.7) 2 (1 to 3)	2.0 (1.6) 2 (0 to 3)	0.642	1.6 (1.4) 1 (0 to 3)	0.8 (1.0) 0 (0 to 2)	0.002
		Not being engaged	0.3 (1.0) 0 (0 to 0)	0.1 (0.6) 0 (0 to 0)	0.179	0.2 (0.8) 0 (0 to 0)	0.1 (0.3) 0 (0 to 0)	0.180	0.4 (1.2) 0 (0 to 0)	0.2 (0.8) 0 (0 to 0)	0.404
			absolute (relative) frequency	absolute (relative) frequency	**Total** ***p***-**value**	absolute (relative) frequency	absolute (relative) frequency	**Older adults** ***p***-**value**	absolute (relative) frequency	absolute (relative) frequency	**Children** *p*-**value**
	**Intergenerational interaction**	Initiating intergenerational interaction	79 (56.4%)	108 (77.1%)	0.001*	47 (60.3%)	55 (70.5%)	0.109	32 (51.6%)	53 (85.5%)	0.000*
			mean (SD) median (IQR)	mean (SD) median (IQR)	**Total** ***p***-**value**	mean (SD) median (IQR)	mean (SD) median (IQR)	**Older adults** ***p***-**value**	mean (SD) median (IQR)	mean (SD) median (IQR)	**Children** ***p***-**value**
**Self-efficacy**	**Observed self-efficacy**	Trying out something new	1.6 (1.0) 1 (1 to 2)	1.5 (0.8) 1 (1 to 2)	0.382	1.7 (1.2) 1 (1 to 2)	1.7 (1.0) 1 (1 to 2)	0.857	1.3 (0.6) 1 (1 to 2)	1.2 (0.5) 1 (1 to 1)	0.157
		Showing confidence in one's abilities	1.6 (1.0) 1 (1 to 2)	1.5 (0.8) 1 (1 to 2)	0.180	1.9 (1.2) 1 (1 to 2)	1.8 (1.0) 1 (1 to 2)	0.604	1.4 (0.6) 1 (1 to 2)	1.2 (0.4) 1 (1 to 1)	0.090
		Coping with demands	1.9 (1.1) 1 (1 to 2)	1.7 (1.0) 1 (1 to 2)	0.018	1.9 (1.2) 1 (1 to 2)	1.8 (1.1) 1 (1 to 2)	0.139	1.8 (0.9) 1 (1 to 3)	1.5 (0.8) 1 (1 to 2)	0.064
		Responding adequately to unexpected situations	1.9 (1.1) 2 (1 to 3)	1.7 (0.9) 1 (1 to 2)	0.063	2.0 (1.2) 2 (1 to 2)	1.7 (0.9) 2 (1 to 2)	0.201	1.8 (0.9) 1 (1 to 3)	1.6 (0.8) 1 (1 to 2)	0.167

Note. bold* = statistically significant results, p < 0.001 (Bonferroni adjusted); used tests: Wilcoxon Signed Rank Tests for ordinal paired data and Mc Nemar test for nominal data (intergenerational interaction); while happy facial expressions increased, the neutral expressions descreased in a similar way due to the fact that the facial expression of each participant was recorded every ten seconds in the assessment (Morita & Kobayashi, 2013) for one minute (maximum score of 6). If happiness significantly increased, the number of other facial expressions recorded would descrease. Instead of being neutral, participants had significantly more happy facial expressions after the intervention

Moreover, a significantly higher number of children (53 out of 62 children; 88.5%; Mc Nemar test: *p* < 0.001) initiated intergenerational contacts at the end of the intervention when compared to baseline (32 children out of 62; 51.6%). Older adults, also showed more self-initiated intergenerational contacts at follow-up compared to baseline (70.5% of older adults initiated intergenerational contact at follow-up, compared to 60.3% at baseline; *p* = 0.109). Some of the secondary outcomes in the areas of engagement/behavior and self-efficacy showed signs of improvement (changes in mean ± SD and median) in children and older adults (Table 3 and Additional file 5).

### Adverse events

In total, 49 adverse events were reported. Of these, 21 were minor, such as restless behavior in children. The major adverse events include one older person and two children falling down without hurting themselves (three falls in total), nine older adults feeling dizzy and having to sit down and another six of them complaining of feeling unwell during a session (solved by making a small break; all older adults participated again in the same session just after the break). The trainers documented six times that one of the older adults had shortness of breath during an activity and four times that one of them was agitated. All adverse events were solved without sequelae.

## Discussion

The present study showed that an intergenerational, psycho-motor activity-based intervention program led to a significantly higher active engagement and well-being as assessed in terms of facial expressions and self-initiated intergenerational contacts after 20 weeks in all participants together and in the kindergarten children separately. Surprisingly, the difference in the older adults was numerically different, but not statistically significant. The qualitative process evaluation, however, showed an overall satisfaction with the program from the perspective of older adults. This was supported by the views of professionals, who also pointed out that the older people had benefitted from the intervention program to a large extent. Furthermore, older adults showed a substantially larger number of actively engaged and happy facial expressions (mean ± SD 2.6 ± 1.8 in older adults compared to 1.6 ± 1.6 in children) already at baseline, leading to a reduced potential for improvement. A possible explanation is the presence of children as such which might immediately increase well-being, active engagement and happiness of older adults from the beginning, whereas children had to become familiar to the intergenerational group sessions.

Furthermore, our study showed that evaluating an intervention prior to full rollout improved the intervention which, in turn, led to a high satisfaction of the participants as well as the professionals. We consider it important in such a mixed methods design that each phase of the evaluation study is informed by the results of the prior phase and builds the basis for the following phase.

Several previous studies assessed the effects of intergenerational interventions only on older adults, but not on the children [19, 20, 22, 23, 48]. The material used in the interventions in our study was specifically selected to induce common activities between older adults and children and some tasks could be solved by older adults and kindergarten children together. Caregivers and parents reported increased self-initiated contacts in both children and older adults outside the intervention session e.g. on the streets, in public transport, in supermarkets and within the families. Similary, participants also pointed out that older adults started to talk to children in the institution or on the street and visited the kindergarten spontaneously after the program had started.

A user-centred development and high feasibility are basic prerequisites for a successful intervention. We therefore used a complex process evaluation design, including qualitative methods, with the intention to support the development and adaptation of the intervention program as close as possible to the needs and requirements of the target groups. Based on the qualitative results in the pilot phase, the rollout phase could be designed in a way to target the needs of all stakeholders. As described above, the qualitative data allowed us more detailed and deeper interpretation of the quantitative results. Using qualitative and quanitative methods in form of a triangulation was essential in our study. We did not only observe potential effects and outcomes, but also most importantly influence the process of development, adaptation to the user-needs and the implementation of an intergenerational intervention. While concerns were raised during the qualitative process evaluation in the pilot phase and reported back to the intervention team, only positive feedback was recorded in the final process evaluation after the rollout.

Strengths of our study are the real life setting and the inclusion of a diversity of people. We included older adults in different living conditions (longterm care and daycare), with different physical, psychosocial and cognitive status including limited mobility (wheel chair or other walking aids), with a wide range of age (54–96 years of age), as well as kindergarten children (2–7 years of age) from different socio-cultural backgrounds to show if the intergenerational intervention program might also work under normal conditions in daily life. Older adults who had looked critically at limited German language skills in some children at the beginning of the study, were convinced at the study’s end that a proper communication was possible even with children whose native language  was different from German. In that sense, limited mobility and possible cognitive impairments of older adults were no reasons for not getting in contact for the group of children.

Despite the schedule of other group sessions at the institutions, the participants perceived the intervention in our study as “something different” from what has been offered so far. This may be due to the facts that the intervention program in our study targeted children and older adults equally, that an activity-inducing material was offered with a task to achieve what could ideally be done by children and older adults together (sharing a common goal) and by introducing material that induces physical activity without repetitive exercises. These results may have led to a higher acceptance and better implementation of the intervention program.

Some studies report that the preschoolers’ attitudes about aging changes in a negative way, when spending time with older adults [49]. However, in contrast and according to the works of Allport [9], and Aday [10] specifically the children’s self-initiated contacts and the number of happy facial expressions increased significantly in our study.

### Limitations of the study

A main aim of our study was to support the development and adaptation of the intervention program. Therefore, the focus was placed on a continuous process evaluation with a feedback in different phases of the project to be able to incorporate this into building up a perfectly targeted intervention to the needs of the participants. A limitation, however, was the lack of a control group. Future studies with a randomized controlled design could be planned based on the results of our study and could show potential effects in comparison of intervention to the control group. Sample sizes for future studies can be based on our effect sizes.

During the rollout some participants dropped out and were replaced by others to keep the group sizes constant. Although this replacement was essential for the intergenerational activities to take place, this is a limitation regarding the data analysis of our study as surrogates participated in a numerically lower number of intervention sessions when compared to the other participants. In the present study, professionals working at the participating institutions were responsible for the recruitment of the participants. In case of any problems, they were offered a support service to facilitate participant recruitment according to the study protocol. As the professionals who performed the assessments observed every group session, the attendance of these people was nothing special for the children and older adults. For this reason, we assume that it was unlikely that participants were just acting because they were being observed.

Furthermore, another assessment might have brought out additional effects for older adults. However, we used assessments that were already established in the literature in similar studies [16, 45]. An important argument for choosing these assessments and looking at happy facial expression was that older adults with and without cognitive impairments, as well as kindergarten children would be able to express their joy and satisfaction in that way. A limitation of this assessment, however, might be that facial expression might differ between individuals. Another limiting factor might have been, that self-efficacy was assessed by professionals only. However, due to the age of the participants and the potentially impaired cognitive abilities some of them might have had, we decided not to use self-reported assessments after an initial pilot test (e.g. self-reported self-efficacy).

### Implications and future directions

Concerning sustainability and transfer aspects of the project, most institutions decided to carry on a similar intergenerational intervention program in the future. In addition, other institutions decided to send some of their professionals and caregivers to be trained in the principles of the intervention program. On the basis of findings from this study further research is recommended to investigate the effectiveness of intergenerational intervention programs based on psycho-motor activity using a randomized controlled trial design including long time follow-up. Furthermore, additional outcomes could be of interest, like possible changes in physical functioning in both target groups.

## Conclusions

Our study showed that older adults and kindergarten children benefit from an intergenerational intervention program based on psycho-motor activity. The results of the qualitative process evaluation led to substantial adaptations of the intervention program from the perspective of different stakeholders throughout all study phases. Professionals in geriatric institutions and kindergartens could facilitate interactions between members of the different generations by offering an intergenerational intervention program based on psycho-motor activities in the future.

## Additional files

Additional file 1: Overview about the content of the intervention program and the activity-inducing material used. (DOCX 14 kb)

Additional file 2: Interview questions for the needs assessment. (DOCX 14 kb)

Additional file 3: Interview questions for the pilot and the rollout phases. (DOCX 16 kb)

Additional file 4: Baseline characteristics of start-to-end participants and surrogates. (DOCX 14 kb)

Additional file 5: Quantitative dataset provided in an anonymized form. (XLSX 42 kb)

## Abbreviations

IQR: Interquartile range
*p*: *p*-value
r: Effect size
RCTs: Randomized controlled trials
SD: Standard deviation
SPSS: Statistical Package for Social Sciences
