# Supplementary material for: An intergenerational program based on psycho-motor activity promotes well-being and interaction between preschool children and older adults: results of a process and outcome evaluation study in Austria
Source: BMC Public Health. 2019 Mar 1;19:254. doi: 10.1186/s12889-019-6572-0 (PMC6397484; doi:10.1186/s12889-019-6572-0)
Supplement: Supplementary file 1 — Overview about the content of the intervention program and the activity-inducing material used. (DOCX 14 kb) [file 12889_2019_6572_MOESM1_ESM.docx]

**Additional file 1** Overview about the content of the intervention program and the activity-inducing material used

| **Session** | **Free and creative usage of …** |
| --- | --- |
| 1 | **Newspapers**  Tearing and folding it, place it on the ground, forming balls for a “snow” ball competition |
| 2 | **Newspapers and tape**  Building a huge sheet of newspapers and use it as a tunnel or for wrapping people |
| 3 | **Cups and plates made of plastic**  Piling them, sliding and pushing them across the floor, building high towers |
| 4 | **Cups and plates made of plastic and ping pong balls**  Additional ideas of what to do with cups, plates and ping pong balls |
| 5 | **Different kinds and sizes of balls**  Playing with them and feeling the differences in weight, surface and size |
| 6 | **Different kinds and sizes of balls and different targets**  Throwing balls towards different targets, e.g.: tunnels, umbrellas hanging the other way round, boxes |
| 7 | **Thin tubes**  Forming different figures, put them together, talking directly into it and listening to others, generating noises |
| 8 | **Thin tubes and marbles**  Building a marble run |
| 9 | **Ropes**  Skipping, knotting, swinging and throwing the ropes |
| 10 | **Ropes and furniture, e.g. chairs and tables**  Building structures to climb over and crawl under it |
| 11 | **Sponges**  Sorting them according to their color and form, cleaning, building paths and walking on them, using them as building blocks |
| 12 | **Sponges and rackets (made of clothes hangers and tights)**  Inventing different plays with the material |
| 13 | **Different kinds of paper**  Tearing and folding it, crumpling it and feeling the differences in the character |
| 14 | **Different kinds of paper and targets**  Making paper airplanes and playing with them |
| 15 | **Plastic threads**  Laying many different patterns, decorating oneself and/or each other, decorating the room |
| 16 | **Plastic threads and clothes hangers**  Building different pieces of art |
| 17 | **Hair ties**  Sorting them, building long necklaces, putting it over fingers, wrists and ankles |
| 18 | **Hair ties and so-called XXL drinking straws** (70cm long)  Inventing different plays with the material |
| 19 | **Balloons**  Balancing them with different parts of the body, playing together, making different noises, holding them in the air as long as possible |
| 20 | **Balloons and fly swatters**  Passing the balloons, targeting different objectives |

*Note: All offered activities should be equally attractive for female and male participants and for older adults and children to the same extent as well as accepted by different cultures. Each material was introduced once; in the subsequent session, the same material was used again (sequence, repetition) in combination with another material (new challenge).*
