# Supplementary material for: An intergenerational program based on psycho-motor activity promotes well-being and interaction between preschool children and older adults: results of a process and outcome evaluation study in Austria
Source: BMC Public Health. 2019 Mar 1;19:254. doi: 10.1186/s12889-019-6572-0 (PMC6397484; doi:10.1186/s12889-019-6572-0)
Supplement: Supplementary file 2 — Interview questions for the needs assessment. (DOCX 14 kb) [file 12889_2019_6572_MOESM2_ESM.docx]

**Additional file 2** Interview questions for the needs assessment

| **Older adults** |
| --- |
| **What comes first to your mind when thinking about kindergarten children?**  **What memories do you have of your own childhood?**  Promts:  What did you particularly like to do?  How was the contact with your own grandparents?  Were there other older people who were important to you?  **Do you now have grandchildren or great-grandchildren?**  Promts:  (If grandchildren yes) What do you prefer to do with them (when they come to visit you)?  What do they like to do with you?  **Do you do physical exercise in your daily life? If yes, on a regular basis? What kind of physical exercise do you do in your everyday life?**  **What do you think of doing activities together with young children?**  Promts:  Would you like to participate in joint activities/group sessions of young children and other older adults?  Is there something you would not like to have in such a joint activity?  What would be the benefit for the children?  Would there be any benefit for older adults?  **Are there any issues that need to be considered when planning joint activities for older adults and young children?** |
| **Children** |
| **Do you know older adults?**  Promts:  How well do you know them?  How often do you see them?  **Which activities do you like to do with them?**  Promts:  What do they enjoy most?  Why do you thing, do older adults like to play with young children?  **Is there something to keep in mind, when playing with older people?**  **What do you wish for once you are old?** |
| **Professionals and parents** |
| **What comes to your mind spontaneously about the relationships between kindergarten children and older people?**  **In the project we are talking about, we will develop and test an intervention with psycho-motor activities. What do you associate with the term “psycho-motor activities”?**  **What expectations do you have on the joint sessions that children and older adults will have?**  Promts:  What do you think will work out well?  Is there anything else to be considered?  **What organizational things need to be considered?** |
