# Supplementary material for: An intergenerational program based on psycho-motor activity promotes well-being and interaction between preschool children and older adults: results of a process and outcome evaluation study in Austria
Source: BMC Public Health. 2019 Mar 1;19:254. doi: 10.1186/s12889-019-6572-0 (PMC6397484; doi:10.1186/s12889-019-6572-0)
Supplement: Supplementary file 3 — Interview questions for the pilot and the rollout phases. (DOCX 16 kb) [file 12889_2019_6572_MOESM3_ESM.docx]

**Additional file 3** Interview questions for the pilot and the rollout phases

| **Older adults** |
| --- |
| **What were your experiences with the intergenerational joint sessions? What are the first things coming to your mind?**  **Was it possible to get in touch with the children? How did you experience it?**  **Which exercises or activities did you like most?**  **Where there any exercises or activities that you did not like? Please explain.**  **Which of the various material did you like?**  **Besides the trainers, there was always someone of your institution and a kindergarten teacher present. How did you experience their presence?**  Promt:  Was it important for you?  **How where the joint activities organised from your perspective?**  Promt:  Did the joint sessions start on time?  **Was an hour duration for a joint session ok from your perspective? Longer/shorter better?**  **Did you regularly take part in the joint sessions?**  Promts:  If yes, was there any support provided for ensuring your participation?  If no: is there anything that might have helped you to participate regularly?  **Is there anything else about this intergenerational group offer that you would like to note?** |
| **Children** |
| **You recently participated in joint activities with older adults. What was it like? Please, tell me about your experiences. Who wants to start?**  **Did you like being together with the older adults?**  Promts:  Which sessions/material did you like most?  Was there something you did not like?  **Did you always take part?**  Promts:  If no: why?  What should have been different?  **X [name of kindergarten teachers] was always there. Did you like that?**  Promts:  If yes: why was that important for you?  If no: why?  **Did you have to wait until the sessions started?**  **Do you still remember the older adults? Do you remember a person particularly well? Why?**  **Will you meet them again?**  Promts:  If yes: when and where?  **Anything else you want to tell me that I haven't asked yet?** |
| **Professionals and parents** |
| **What were your experiences with the psycho-motor activity joint sessions for older adults and kindergarten children?/ What did your children tell about the offer?**  **Were your expectations of the intergenerational intervention fulfilled? What insights have you gained from the project so far?**  **How did you experience the presence of the kindergarten teacher and the caregiver, respectively?**  **Did the older adults/children regularly take part in the group offer?**  Promts:  If yes, was there any support?  If no: What were the reasons for not participating?  What do you think could increase the commitment to participate in the offer?  **What do you think about one hour duration for the intergenerational activity?**  **Is there anything else about these joint sessions that you would like to bring up?** |
