# Supplementary material for: An intergenerational program based on psycho-motor activity promotes well-being and interaction between preschool children and older adults: results of a process and outcome evaluation study in Austria
Source: BMC Public Health. 2019 Mar 1;19:254. doi: 10.1186/s12889-019-6572-0 (PMC6397484; doi:10.1186/s12889-019-6572-0)
Supplement: Supplementary file 4 — Baseline characteristics of start-to-end participants and surrogates. (DOCX 14 kb) [file 12889_2019_6572_MOESM4_ESM.docx]

**Additional file 4** Baseline characteristics of start-to-end participants and surrogates

| **Outcome** | **Assessment** | **Categories/Items** | **Start-to-end participants**  **Total (n=100) baseline**  mean (SD) median (IQR) | **Surrogate participants Total (n=20) follow-up**  mean (SD) median (IQR) | **Total p-value** |
| --- | --- | --- | --- | --- | --- |
| **Active engagement** | **Facial expression** (primary outcome) | Happy/smiling | 2.1 (1.8) 2 (0 to 3) | 2.6 (1.7) 3 (1 to 4) | 0.243 |
|  |  | Neutral | 2.7 (1.8) 4 (2 to 5) | 2.8 (1.7) 3 (2 to 4) | 0.073 |
|  |  | Lethargic | 0.2 (0.8) 0 (0 to 0) | 0.4 (1.2) 0 (0 to 0) | 0.359 |
|  |  | Grumpy | 0.0 (0.3) 0 (0 to 0) | 0.1 (0.5) 0 (0 to 0) | 0.346 |
|  | **Engagement/ behavior** | Participating actively | 4.0 (1.6) 4 (3 to 5) | 3.6 (2.2) 4 (1.5 to 5) | 0.729 |
|  |  | Paying attention/listening | 1.8 (1.5) 2 (0 to 3) | 2.0 (2.0) 1.5 (0.25 to 2.75) | 0.966 |
|  |  | Being not engaged | 0.2 (1.0) 0 (0 to 0) | 0.4 (1.5) 0 (0 to 0) | 0.903 |
|  |  |  | absolute (relative) frequency | absolute (relative) frequency | **Total p-value** |
|  | **Intergenerational interaction** | Initiating intergenerational interaction | 59 (59%) | 9 (45%) | 0.763 |
|  |  |  | mean (SD) median (IQR) | mean (SD) median (IQR) | **Total p-value** |
| **Self-efficacy** | **Observed self-efficacy** | Trying out something new | 1.4 (0.8) 1 (1 to 2) | 1.9 (1.3) 1 (1 to 3) | 0.189 |
|  |  | Showing confidence in its own abilities | 1.5 (0.9) 1 (1 to 2) | 2.1 (1.3) 2 (1 to 3) | 0.069 |
|  |  | Coping with demands | 1.7 (1.0) 1 (1 to 2) | 2 (1.2) 2 (1 to 2) | 0.367 |
|  |  | Responding adequately to unexpected situations | 1.8 (1.0) 1 (1 to 2) | 2 (1.3) 1.5 (1 to 3) | 0.716 |
